# Lifecourse socioeconomic circumstances and multimorbidity among older adults

**DOI:** 10.1186/1471-2458-11-313

**Published:** 2011-05-14

**Authors:** Reginald D Tucker-Seeley, Yi Li, Glorian Sorensen, SV Subramanian

**Affiliations:** 1 Center for Community Based Research, Dana-Farber Cancer Institute, 450 Brookline Ave, Boston, MA, 02215 USA; 2 Department of Society, Human Development, and Health, Harvard School of Public Health, 677 Huntington Ave, Boston, MA, 02115 USA; 3 Department of Biostatistics and Computational Biology, Dana-Farber Cancer Institute, 450 Brookline Ave, Boston, MA, 02215 USA; 4 Department of Biostatistics, Harvard School of Public Health, 677 Huntington Ave, Boston, MA, 02115 USA

## Abstract

**Background:**

Many older adults manage multiple chronic conditions (i.e. multimorbidity); and many of these chronic conditions share common risk factors such as low socioeconomic status (SES) in adulthood and low SES across the lifecourse. To better capture socioeconomic condition in childhood, recent research in lifecourse epidemiology has broadened the notion of SES to include the experience of specific hardships. In this study we investigate the association among childhood financial hardship, lifetime earnings, and multimorbidity.

**Methods:**

Cross-sectional analysis of 7,305 participants age 50 and older from the 2004 Health and Retirement Study (HRS) who also gave permission for their HRS records to be linked to their Social Security Records in the United States. Zero-inflated Poisson regression models were used to simultaneously model the likelihood of the absence of morbidity and the expected number of chronic conditions.

**Results:**

Childhood financial hardship and lifetime earnings were not associated with the absence of morbidity. However, childhood financial hardship was associated with an 8% higher number of chronic conditions; and, an increase in lifetime earnings, operationalized as average annual earnings during young and middle adulthood, was associated with a 5% lower number of chronic conditions reported. We also found a significant interaction between childhood financial hardship and lifetime earnings on multimorbidity.

**Conclusions:**

This study shows that childhood financial hardship and lifetime earnings are associated with multimorbidity, but not associated with the absence of morbidity. Lifetime earnings modified the association between childhood financial hardship and multimorbidity suggesting that this association is differentially influential depending on earnings across young and middle adulthood. Further research is needed to elucidate lifecourse socioeconomic pathways associated with the absence of morbidity and the presence of multimorbidity among older adults.

## Background

Chronic conditions generally cluster and individuals with one chronic condition are likely to have other conditions as well. As such, many older adults now manage two or more chronic conditions at the same time (i.e. multimorbidity)[1,2]; and evidence suggests that the presence of multimorbidity is expected to continue to rise [3-5]. The influence of childhood material conditions and lifetime earnings on multimorbidity has yet to be investigated. Considering the impending shift in the US population demographic as the baby-boomers reach age 65, which will yield an elderly population characterized by declining death rates, increasing life expectancy, and increasing health care costs,[6,7] an understanding of the correlates of multimorbidity in older adults is a crucial issue for population health research [8,9].

### Lifecourse financial/economic conditions

A substantial literature exists documenting the positive association between childhood SES and adult health[10]; specifically, low childhood socioeconomic status (e.g. parental occupation, parental educational attainment) has been linked to heart disease,[11] stroke [12], diabetes [13], and some cancers [14] among adults. A few lifecourse epidemiology studies have broadened the notion of childhood socioeconomic status (SES) to include specific financial hardships present in the socioeconomic environment during childhood [15-17]. For example, Lundberg (1997) found that respondents growing up in a family that experienced economic difficulties had an increased risk for circulatory illness in adulthood, even after controlling for demographic and socioeconomic characteristics and baseline health [17]. Additionally, Moody-Ayers, et al (2007) showed an independent effect of childhood financial hardship on functional limitations among older adults over and above the influence of adult SES and demographic characteristics [18].

Financial hardships in childhood can greatly influence subsequent access to financial/economic resources,[19,20] which in turn affects the trajectory of health outcomes throughout the lifecourse [21]. As such, SES over the lifecourse has been shown to be more strongly associated with adult disease outcomes than an assessment of SES for a particular point in time [22,23]. In particular, cumulative disadvantage or low lifecourse SES has been shown to be positively associated with morbidity and mortality [24-27]. However, the relationship among childhood financial hardship, earnings across adulthood, and multimorbidity remains to be investigated; in particular, to our knowledge no study has been conducted to determine the association of these factors among older adults.

The aims for the present study were threefold. The first aim was to determine the association between childhood financial hardship and multimorbidity adjusting for demographic and socioeconomic characteristics. The second aim was to determine the association between lifetime earnings and multimorbidity; and the third aim was to determine if lifetime earnings act as an effect modifier in the association between childhood financial hardship and adult multimorbidity.

## Methods

### Data source

The Health and Retirement Study (HRS) is a national longitudinal study of the economic, health, marital, family status, and public/private support systems of older Americans funded by the National Institute on Aging and the Social Security Administration and conducted by the Institute for Social Research Survey Research Center at the University of Michigan [28]. The HRS uses a national multistage area probability sample of households in the U.S., with oversamples of Blacks, Hispanics, and residents from the state of Florida. Details of the HRS data collection methods are described elsewhere [29]. We used respondents from the 2004 wave of the HRS who signed consent forms for their social security earnings records to be linked to their HRS records (N = 7,792). To determine lifetime earnings of the respondents, we linked the 2004 HRS public use dataset to the 2004 Permissions: Summary Earnings Information Restricted data set provided by the HRS. The 2004 Permissions data set includes earnings data for HRS respondents from 1951 to 2003. Subjects were dropped from analyses if they were under age 50 (N = 369), if they were missing data on the childhood financial hardship variable (N = 37), and if they were missing data on the multimorbidity variable (N = 61) or the socioeconomic variables (N = 20). The final sample used for analysis was N = 7,305. The Institutional Review Board (IRB) at the University of Michigan approved the Health and Retirement Study and the Office of Human Research Administration at the Harvard School of Public Health approved this study.

### Outcome variable

*Multimorbidity *was operationalized as the count of six chronic conditions reported in the 2004 HRS. The chronic conditions of interest were cancer, heart disease, lung disease, stroke, diabetes, and hypertension. Chronic conditions were assessed in the HRS by a series of questions where respondents were asked if a doctor had ever told them if they had one of these diseases.

### Primary predictor variables

We operationalized *childhood financial hardship *with the dichotomous (yes/no) question from the HRS: 1) While you were growing up, before age 16, did financial difficulties ever cause you or your family to move to a different place?

We used average annual lifetime earnings during young and middle adulthood to operationalize *lifetime earnings*. Average annual lifetime earnings were calculated as the mean annual earnings of the respondent from age 20 to age 50. We selected up to age 50 because this is the age of eligibility for the HRS, thus all respondents would potentially have earnings up to age 50. Years with zero earnings were excluded from the calculation of average annual lifetime earnings to avoid misclassifying respondents who may temporarily leave the labor market (e.g. respondents with child-rearing responsibilities) [30]. Earnings were inflation-adjusted to 2003 US dollars using the consumer price index for urban consumers (CPI-U 1951-2003) [31]. For ease of interpretation, we divided average annual earnings by $10,000 so that changes in the outcome would be associated with a $10,000 change in average annual earnings.

### Covariates

Educational attainment was used as an indicator of adult SES [32]. It was grouped into three categories: less than high school, high school diploma/GED, and some college or more.

Demographic characteristics such as gender, race (white/non-white), ethnicity (Hispanic/non-Hispanic), and age were assessed by self-report in the HRS. Tertiles were created for the age variable: 50-59, 60-69, and 70 and older for univariate and bivariate analysis.

### Statistical analysis

In order to test the association among childhood financial hardship, average annual lifetime earnings, and multimorbidity, bivariate and multivariable tests were performed. Bivariate analyses using Wilcoxon-Mann-Whitney tests were conducted to determine differences in multimorbidity between demographic categories and Kruskall-Wallis tests were conducted to determine differences in multimorbidity across SES categories. Additionally, unadjusted rate ratios were estimated with robust standard errors to quantify differences across demographic and SES categories in multimorbidity.

To take into consideration the count nature of the outcome variable and the substantial number of respondents reporting no chronic conditions (*Y = 0*), zero-inflated Poisson (ZIP) regression was used [33] (30% of respondents in this sample reported no chronic conditions). The ZIP model assumes that the respondents potentially come from two different distributions: one distribution that is indeed likely to have a zero count (no disease), and another distribution that follows the Poisson distribution (count of the six chronic conditions measured in this study) [34]. The ZIP model is a two-part model represented as: 1) P (*y*_*i*_|*x*_*i*_) = *p*_*i *_+ (1 - *p*_*i*_) exp(-*μ*_*i*_) when *y_i _*= 0 and; 2)  when *y_i_*≥1 [35]. The first part represents a logistic model that estimates the change in the log-odds of reporting no chronic conditions (absence of morbidity) for each one-unit change in the independent variable, and the second part represents a Poisson model that estimates the percentage change in the number of chronic conditions for a one-unit change in the independent variable. The justification for this approach is that the factors associated with having one or more chronic conditions might be differentially associated with the absence of morbidity (reporting no chronic conditions).

SAS 9.2^© ^was used for model building. Following bivariate analysis, we developed unadjusted models to evaluate the association between childhood financial hardship and multimorbidity in 2004 (Model 1). Next, we adjusted for demographic (Model 2) and socioeconomic characteristics (Model 3). We then developed unadjusted models to evaluate the association between lifetime earnings and multimorbidity in 2004 (Model 4). Next, we adjusted this model for demographic (Model 5) and socioeconomic characteristics (Model 6). We then entered average annual lifetime earnings into Model 3 to evaluate the influence of lifetime earnings on the childhood financial hardship adjusted model (Model 7). To determine if the association between childhood financial hardship and multimorbidity was modified by lifetime earnings, we entered an interaction term (lifetime earnings × childhood financial hardship) into the model (Model 8) [36].

## Results

Table 1 lists the descriptive statistics. The average age for the full sample was 65 years old. Nineteen percent of the sample reported childhood financial hardship. The mean number of chronic conditions was 1.24, with 30% (N = 2,193) reporting no chronic conditions. The mean number of chronic conditions for those reporting childhood financial hardship was 1.35 and the mean number of chronic conditions for those reporting no childhood financial hardship was 1.20. The median average annual lifetime earnings up to age 50 (CPI-U adjusted) was $19,410 with an inter-quartile range of $20,235 (see Table 1).

**Table 1:** Frequency distributions for childhood financial hardship (CFH) and chronic conditions and bivariate associations between demographic and socioeconomic characteristics and multimorbidity

			*Childhood Financial Hardship*		*Number of Chronic Conditions (Multimorbidity)*					
	*N*	*%*	*CFH = 0*	*CFH = 1*	*0*	*1*	*2*	*3*	*4 or more*	*P-VALUE*

Total	7,305		5,946	1,359	2,193	2,524	1,580	730	278	
**Gender**										p < .001
*Female*	3,916	53.61	3,244	672	1,238	1,382	769	374	153	
*Male*	3,389	46.39	2,702	687	955	1,142	811	356	125	
**Race**										p < .001
*White*	5,907	80.87	4,827	1,080	1,861	2,053	1,241	550	202	
*Non-white*	1,397	19.13	1,118	279	331	471	339	180	76	
**Hispanic Ethnicity**										p = .12
*Hispanic*	740	10.13	570	170	233	260	162	67	18	
*Non-Hispanic*	6,565	89.87	5,376	1,189	1,960	2,264	1,418	663	260	
**Age**										
*50-59*	2,004	27.43	1,615	389	921	669	283	104	27	p < .001
*60-69*	3,218	44.05	2,661	557	866	1,169	707	338	138	
*≥ 70*	2,083	28.51	1,670	413	406	686	590	288	113	
**Education**										p < .001
*Less than High School*	1,705	23.34	1,285	420	367	537	435	238	128	
*High School/GED*	2,410	32.99	2,007	403	700	850	537	245	78	
*Some College or more*	3,190	43.67	2,654	536	1,126	1,137	608	247	72	
Median AVGEARN_LIFETIME^†^	$19,410		$19,488	$19,138						
Inter-quartile Range AVGEARN_LIFETIME	$20,235									

^† ^AVGEARN_LIFETIME = Average annual lifetime earnings (age 20-50) which are adjusted using the CPI-U to account for inflation

Bivariate analysis suggests differences in multimorbidity across all demographic and socioeconomic categories except Hispanic ethnicity (see Table 1). Unadjusted rate ratios showed that men reported slightly higher multimorbidity than women. In particular, the expected number of chronic conditions in men was 8% higher than in women. For non-white respondents, the unadjusted expected number of chronic conditions was 21% higher compared to white respondents. The unadjusted expected number of chronic conditions for those age 60-69 and 70 and older was 56% and 85%, respectively, greater than the respondents age 50-59. The unadjusted expected number of chronic conditions across education categories was 16% higher for those with educational attainment of high school/GED and 46% higher for those with less than high school compared to the some college or more group of respondents. No statistically significant differences in multimorbidity were noted in Hispanic ethnicity; therefore, ethnicity was not included in subsequent models (see Table 2).

**Table 2:** Unadjusted rate ratios of bivariate associations between the demographic and socioeconomic variables and multimorbidity

	*Multimorbidity*
**Gender**	
*Female*	1.00
*Male*	1.08** (1.03, 1.12)
**Race**	
*White*	1.00
*Non-white*	1.21*** (1.15, 1.26)
**Hispanic Ethnicity**	
*Hispanic*	.94 (.87, 1.00)
*Non-Hispanic*	1.00
**Age**	
*50-59*	1.00
*60-69*	1.56*** (1.47, 1.65)
*≥ 70*	1.85*** (1.74, 1.96)
**Education**	
*Less than High School*	1.46*** (1.39, 1.53)
*High School/GED*	1.16*** (1.11, 1.22)
*Some College or more*	1.00

p < .05; ** p < .01; *** p < .001

### Multivariable analysis: childhood financial hardship and multimorbidity

The logistic portion of the unadjusted and adjusted ZIP regression models showed that childhood financial hardship was not significantly associated with the absence of morbidity (see Table 3, models 1-3, logistic portion). The Poisson portion of the unadjusted ZIP model showed that the expected number of chronic conditions for those reporting childhood financial hardship was 1.11 (CI: 1.04, 1.19) times that of those not reporting childhood financial hardship. After adjusting for gender, age, and race, the difference in expected number of chronic conditions between those reporting childhood financial hardship and those not reporting childhood financial hardship did not change substantially (exp(β) = 1.10; CI: 1.04, 1.16). After adjusting for educational attainment, the expected number of chronic conditions for those reporting childhood financial hardship was reduced to 1.08 (CI: 1.02, 1.14) times greater than for those not reporting childhood financial hardship (See Table 3, models 1-3, Poisson portion).

**Table 3:** Unadjusted and adjusted exponentiated parameter estimates and confidence intervals for the zero-inflated Poisson regression models testing the association between childhood financial hardship and multimorbidity

Logistic portion of the ZIP model			
	*Model 1*	*Model 2*	*Model 3*
Childhood financial hardship^†^	.30 (.004, 46.63)	.39 (.10, 1.52)	.43 (.11, 1.60)
Gender *(female is reference)*		.92 (.46, 1.87)	1.00 (.49, 2.04)
Age		.77*** (.69, .82)	.78*** (.68, .82)
Race *(white is reference*)		4.03 (.74, 22.01)	.28 (.05, 1.22)
Educational Attainment *(less than high school)*			
High School/GED			1.79 (.74, 4.34)
Some College or more			.79 (.29, 2.16)
Poisson Portion of the ZIP model			
	*Model 1*	*Model 2*	*Model 3*

Childhood financial hardship^†^	1.11** (1.04, 1.19)	1.10*** (1.04, 1.16)	1.08** (1.02, 1.14)
Gender *(female is reference)*		.36 (.10, 1.39)	.41 (.11, 1.50)
Age		1.02*** (1.02, 1.03)	1.02*** (1.02, 1.03)
Race *(white is reference group*)		.73 (.37, 1.45)	1.14 (.56, 2.32)
Educational Attainment *(less than high school)*			
High School/GED			.21* (.05, .93)
Some College or more			1.52 (.63, 3.67)

p < .05; ** p < .01; *** p < .001; ^† ^Childhood financial hardship was measured by a question, "While you were growing up, before age 16, did financial difficulties ever cause you or your family to move to a different place?"

### Multivariable analysis: lifetime earnings and multimorbidity

The logistic portion of the ZIP models showed that lifetime earnings and the absence of morbidity were only associated in the unadjusted model (exp(β) = .55; CI: .28, .91; see Table 4, models 4-6, logistic portion). The Poisson portion of the unadjusted ZIP model showed a 7% decrease in the expected number of chronic conditions for each $10,000 increase in average annual lifetime earnings (see Table 4, model 4). After adjusting for gender, age, and race the percent decrease was unchanged at 7%. However, after adjusting for educational attainment the percent decrease in the expected number of chronic conditions was reduced to 5% for each $10,000 increase in average annual earnings (see Table 4, model 6, Poisson portion).

**Table 4:** Unadjusted and adjusted exponentiated parameter estimates and confidence intervals for the zero-inflated Poisson regression models testing the association between lifetime earnings and multimorbidity

Logistic portion of the ZIP model			
	*Model 4*	*Model 5*	*Model 6*
Lifetime Earnings^§^	.55* (.28, .91)	1.08 (.81, 1.42)	1.10 (.80, 1.51)
Gender		1.15 (.50, 2.64)	1.16 (.50, 2.71)
Age		.77*** (.67, .83)	.76*** (.66, .82)
Race *(white is reference*)		.35 (.08, 1.45)	.32 (.08, 1.37)
Educational Attainment *(less than high school is reference)*			
High School/GED			2.03 (.73, 5.69)
Some College or more			.84 (.27, 2.55)
Poisson Portion of the ZIP model			
	*Model 4*	*Model 5*	*Model 6*

lifetime earnings^§^	.93*** (.91, .95)	.93*** (.91, .95)	.95*** (.93, .98)
Gender		1.14*** (1.08, 1.20)	1.11*** (1.05, 1.17)
Age		1.02*** (1.02, 1.03)	1.02*** (1.02, 1.03)
Race *(white is reference*)		1.22 (.54, 2.78)	1.23 (.53, 2.83)
Educational Attainment *(less than high school is reference)*			
High School/GED			.26 (.06, 1.06)
Some College or more			1.76 (.63, 4.89)

p < .05; ** p < .01; *** p < .001; ^§ ^Lifetime earnings was operationalized as average annual income from 1951 up to age 50 of the respondent CPI-U adjusted

### Multivariable analysis: childhood financial hardship and lifetime earnings mutually adjusted and interaction term added

Including childhood financial hardship and lifetime earnings into the ZIP model (see Table 5, model 7) showed a slight change in the childhood financial hardship rate ratio from the fully adjusted model (see Table 3, model 3) of 1.08 to 1.07, but the rate ratio for lifetime earnings from the fully adjusted model (see Table 4, model 6) was unchanged (see Table 5, model 7). The childhood financial hardship × average annual lifetime earnings interaction term was statistically significant (exp(β) = .95; CI = .91, .99); and with the inclusion of this interaction term the rate ratio for childhood financial hardship increased (exp(β) = 1.19; CI: 1.07, 1.32) and the rate ratio for lifetime earnings changed by 2% (exp(β) = .97; CI = .94, .99; see Table 5, model 8).

**Table 5:** Fully adjusted exponentiated parameter estimates and confidence intervals for zero-inflated Poisson regression models testing the association among childhood financial hardship, lifetime earnings, and multimorbidity, and the interaction between childhood financial hardship and lifetime earnings

Logistic portion of the ZIP model		
	*Model 7*	*Model 8*
Childhood financial hardship^†^	.40 (.09, 1.71)	.27 (.04, 2.05)
Gender *(female is reference)*	1.16 (.50, 2.70)	1.05 (.47, 2.33)
Age	.76*** (.67, .82)	.75*** (.66, .81)
Race *(white is reference*)	.34 (.10, 1.23)	.34 (.10, 1.15)
Educational Attainment *(less than high school reference)*		
High School/GED	1.91 (.71, 5.11)	1.49 (.61, 3.67)
Some College or more	.67 (.24, 1.89)	.62 (.22, 1.70)
Lifetime Earnings^§^	1.03 (.73, 1.42)	1.06 (.77, 1.44)
Childhood financial hardship X Lifetime earnings		1.35 (.35, 3.36)
Poisson Portion of the ZIP model		
	*Model 7*	*Model 8*

Childhood financial hardship^†^	1.07* (1.01, 1.13)	1.19*** (1.07, 1.32)
Gender *(female is reference)*	.41 (.10, 1.74)	.25 (.03, 1.83)
Age	1.02*** (1.02, 1.03)	1.02*** (1.02, 1.03)
Race *(white is reference group*)	1.23 (.53, 2.82)	1.12 (.51, 2.46)
Educational Attainment *(less than high school is reference)*		
High School/GED	.27* (.08, .95)	.27* (.08, .89)
Some College or more	1.66 (.62, 4.41)	1.49 (.61, 3.67)
Lifetime Earnings^§^	.95*** (.93, .97)	.97* (.94, .99)
Childhood financial hardship X Lifetime earnings		.95* (.91, .99)

* p < .05; ** p < .01; *** p < .001; ^† ^Childhood financial hardship was measured by a question, "While you were growing up, before age 16, did financial difficulties ever cause you or your family to move to a different place?" ^§ ^Lifetime Earnings was operationalized as average annual income from 1951 up to age 50 of the respondent CPI-U adjusted

## Discussion

This study investigated the association among childhood financial hardship, lifetime earnings, and multimorbidity in a sample of older adults. Our findings revealed that after controlling for socioeconomic and demographic characteristics, childhood financial hardship was positively associated with a higher number of the chronic conditions. This suggests that over and above the influence of age, race, and educational attainment, childhood financial hardship exerts an influence on the multimorbidity of the six chronic conditions measured in this study for older adults. Our findings also indicate that lifetime earnings was negatively associated with multimorbidity, although the noted association was relatively small. In particular, we showed that as the average annual income during young and middle adulthood increases by $10,000 the number of chronic conditions (as measured in this study) decreases by 5%. Additionally, when we included both childhood financial hardship and lifetime earnings in our models, the association between lifetime earnings and multimorbidity remained unchanged and the association between childhood financial hardship and multimorbidity was only slightly reduced. However, our tests of interactions revealed that lifetime earnings significantly modifies the relationship between childhood financial hardship and multimorbidity. This suggests that the influence of financial hardship in childhood on subsequent multimorbidity may be altered by earnings occurring in young and middle adulthood. More specifically, our findings showed that for older adults experiencing childhood financial hardship an increase by $10,000 in average annual earnings reduces the expected number of chronic conditions by 5%. Lastly, although we were primarily interested in determining the association among childhood financial hardship, lifetime earnings, and multimorbidity over and above the influence of other socioeconomic indicators such as educational attainment, it should be noted that increasing education was not consistently associated with an increase in the count of the six chronic conditions in this study. In particular, educational attainment was not associated with the absence of morbidity; and when compared to the less than high school group, only the high school/GED category showed a protective association with multimorbidity.

We evaluated our hypotheses using ZIP regression; and, the benefit of a ZIP modeling approach is the simultaneous estimation of factors associated with multimorbidity and the absence of morbidity. In fully adjusted models, childhood financial hardship was not associated with the absence of morbidity and lifetime earnings was only associated with the absence of morbidity in unadjusted models. Consistent with the literature, the logistic portion of our ZIP models showed that age was strongly negatively associated with the absence of morbidity [37]. Lastly, the widest confidence intervals were noted in the logistic portion of the childhood financial hardship ZIP models. This may indicate poor model fit and suggests that the potential socioeconomic and demographic factors associated with determining the number of diseases (of those measured in this study) might be substantively different from the factors associated with determining the absence of morbidity.

The lack of an association between the several indicators of socioeconomic circumstances in this study (i.e. childhood financial hardship, lifetime earnings, and educational attainment) and demographic characteristics (i.e. race and gender) and the absence of morbidity was unexpected. Research has shown a negative association between socioeconomic status and morbidity[38,39] and multimorbidity[40,41]; and studies on successful aging (where one component is the absence or low risk of morbidity[42]) have shown that childhood and mid-life socioeconomic circumstances[43], and the stability of financial resources[44] were positively associated with the absence of morbidity in older adults. However, the evidence on the association between socioeconomic factors and successful aging is equivocal[45-47]. It has also been suggested that age effects on self-reported morbidity may overshadow socioeconomic effects[48]. Not surprising, our results show an independent association between the absence of morbidity and age, even when childhood financial hardship and lifetime earnings are included in the model. Demographic characteristics[48] and psychosocial and behavioral factors[49] across the lifecourse may prove more important than socioeconomic factors in determining the absence of morbidity; however, the influence of lifecourse socioeconomic factors on the absence of morbidity requires further exploration to fully elucidate their role in successful aging in general and the absence of morbidity in particular among older adults.

### Childhood socioeconomic condition and adult health

Financial and economic circumstances occurring in childhood and throughout the lifecourse have been shown to affect adult health outcomes [50]. Research shows that antecedents to the socioeconomic gradient in adult health can be seen in the socioeconomic environment in childhood [51]. As such, the childhood environment can, 'cast long shadows forward' on future health outcomes [52]; that is, the financial and economic circumstances occurring and accumulating throughout the lifecourse can determine and influence the health trajectory of the individual [53,54].

Additionally, it has been suggested that many chronic diseases share common risk factors [55]. In particular, current low socioeconomic status and disadvantage accumulated across the lifecourse have been shown to be significant pathways to many chronic conditions [25,56]; and, socio-environmental factors experienced at various stages throughout the lifecourse can differentially impact disease etiology [57]. So, even though chronic diseases have long latency periods, research has consistently shown that for many chronic conditions adult and childhood socioeconomic factors can have a considerable impact on health outcomes [58,59]. Our results are consistent with these findings. In particular, our results show that an expanded notion of SES that includes hardships during childhood and earnings throughout adulthood may also uncover possible associations between socioeconomic conditions and adult health. Our findings also show that a possible modifier of the relationship between childhood financial conditions and the number of adult chronic conditions that deserves further attention is earnings during young and middle adulthood.

## Limitations

There are limitations to the present study. First, respondents in the HRS were asked to recall childhood financial hardship experiences; as a result, the measures of childhood financial hardship may be subject to recall bias. Second, our sample only included those individuals who provided permission for their social security records to be linked to their HRS records potentially introducing sample selection bias. However, it has been shown that the HRS respondents who grant permission for their social security were not very much different from those who did not [60]. In addition, the social security data linked to the HRS is capped at the taxable maximum for a given year and only includes earnings subject to Social Security deduction. Next, the literature remains inconsistent in defining multimorbidity [61,62]; for example, some studies define multimorbidity as the count of two or more chronic conditions,[40] others use a combination of specific diseases, and still others use a simple count of chronic conditions reported or obtained from medical records [63,64]. As a result, little guidance is provided in selecting a measure for multimorbidity and the specific diseases such a measure should contain. However, the chronic diseases selected for the present study are consistent with those of other studies of multimorbidity among older adults [21,65]. Lastly, our study is cross-sectional so causal inferences cannot be definitively made regarding the associations noted.

## Conclusions

Multimorbidity has considerable implications for health-related research, health care, and many government systems (e.g. Medicare, Medicaid, and Veterans Administration). Due to the single disease focus in research and health care[66,67], we know relatively little about the correlates of multimorbidity [68,69]. Yet, health care models on the delivery of clinical services for the elderly with multiple chronic conditions have recently been introduced highlighting the impact the growing problem of multimorbidity has on health care delivery [70,71]. However, we know little about the factors that are associated with the various combinations of morbidity experienced by older adults, and such knowledge could potentially improve care and delay mortality for many managing multiple chronic conditions [72]. Additional research focused on the specific socioeconomic factors associated with multimorbidity across the lifecourse can also be used to inform the development of appropriate interventions that target socioeconomic groups at greatest risk for multimorbidity before they enter the health care system. In particular, interventions that target specific socioeconomic pathways might prove useful in helping reduce the burden of multimorbidity; more specifically, interventions that focus on material resources such as reducing hardships during childhood and increasing earnings throughout young and middle adulthood may have a substantial impact on prevention efforts[73] with those at risk for various combinations of chronic disease multimorbidity.

## Competing interests

The authors declare that they have no competing interests.

## Authors' contributions

RDT-S, YL, GS, and SVS conceived of the study; RDT-S and YL developed the analysis plan and performed the statistical analysis; RDT-S, YL, GS, and SVS interpreted the results; RDT-S wrote the first draft of the manuscript. RDT-S, YL, GS, and SVS edited the manuscript. All authors read and approved the final manuscript.

## Pre-publication history

The pre-publication history for this paper can be accessed here:

http://www.biomedcentral.com/1471-2458/11/313/prepub
